# Astragalus *fascicolifolius* manna abortifacient risk and effects on sex hormones in BALB/c mice

**DOI:** 10.37796/2211-8039.1113

**Published:** 2020-12-01

**Authors:** Mehrdad Shahrani, Najmeh Asgharzadeh, Soleiman Kheiri, Roya Karimi, Arezo Sadeghimanesh, Shirin Asgharian, Zahra Lorigooini

**Affiliations:** a Medical Plants Research Center, Basic Health Sciences Institute, Shahrekord University of Medical Sciences, Shahrekord, Iran; b Clinical Biochemistry Research Center, Shahrekord University of Medical Sciences, Shahrekord, Iran

**Keywords:** *Astragalus fascicolifolius* manna, estrogen, progesterone, FSH, LH

## Abstract

**Background:**

*Astragalus fascicolifolius* manna is used to treat different diseases. Because pregnant women tend to use *Astragalus. fascicolifolius* and Iranian traditional medicine emphasizes the abortifacient potential of this plant, this study aimed to investigate *Astragalus fascicolifolius* manna abortifacient property and effects on estrogen, progesterone, LH and FSH levels in BALB/c mice.

**Method:**

This experimental study was conducted with 70 female BALB/c mice assigned to seven groups: Nonpregnant, untreated; nonpregnant, *Astragalus. fascicolifolius* extract (400 mg/kg)-treated; pregnant, *Astragalus. fascicolifolius* extract (400, 800 and 1200 mg/kg)-treated; and two pregnant control groups. On 18 and 19 days of pregnancy, cesarean section performed on mice, resorbed embryos counted; then Follicle-stimulating hormone (FSH), Luteinizing hormone (LH), estrogen and progesterone levels were measured by the ELISA.

**Results:**

*Astragalus. fascicolifolius* extract caused a significant increase abortion in mice. The levels of progesterone, FSH and LH were significantly different among the groups such that mean progesterone level was lower and mean LH and FSH levels were higher in the *Astragalus. fascicolifolius* extract-treated groups than the pregnant, untreated group.

**Conclusion:**

This extract has abortifacient properties and this plant can be used cautiously in pregnancy. Decreasing progesterone, increasing FSH and LH feedback in response to decreased progesterone by this extract is one of the potential mechanisms involved in abortion.

## 1. Introduction

Abortion refers to the spontaneous or deliberate ending of a pregnancy before the fetus has evolved sufficiently to survive. Overall, abortion is the termination of pregnancy before 20 weeks of pregnancy [1-3]. The causes of abortion include embryonic factors including chromosomal abnormalities and abnormal evolution of the zygote, and maternal factors including uterine anatomical abnormalities, immunological factors, coagulation disorders, endocrine factors, infections, environmental factors, physical trauma and cervical failure [4]. Induced abortion is the deliberate termination of pregnancy using medical or surgical procedures before the embryo can survive. The techniques for abortion can be surgical or medical procedures. Surgical techniques include dilatation and curettage, dilatation and discharging, dilatation and withdrawal, menstrual aspiration and laparotomy. In medical techniques, three drugs consisting of antiprogestin mifepristone, antimetabolite methotrexate, and prostaglandin misoprostol are used. These medications cause abortion through increasing uterine contractility [5].

In pregnancy, certain changes occur in the secretion of hormones. Gonadotropin-releasing hormone (GnRH) is released from syncytial trophoblasts to the mother's body fluids. The most important action of this hormone is to prevent the corpus luteum from regression at the end of the menstrual cycle. In pregnancy, the first estrogen is secreted from the corpus luteum and, after placental growth; it is secreted from the placenta. In late pregnancy, estrogen to progesterone ratio increases, which is responsible for increased uterine contractility and the progression of the delivery process. Progesterone is essential for the progression of pregnancy, and during pregnancy, it is first secreted from the corpus luteum and then the placenta [6].

Medicinal plants have been commonly used in Iran and other countries since ancient times. The use of medicinal plants has changed greatly at different times depending on the time requirements [7, 8]. In recent years, the use of medicinal plants has increased sharply[9, 10]. Most pregnant women assume that nature-based medications do not lead to any drug interaction for fetal and maternal and therefore turn to self-medication with them, while they may cause certain fetal and maternal side effects or drug interactions [11, 12]. To consider that, *A. fascicolifolius* manna is used to treat different diseases and may pregnant women consume it to treat the problems during pregnancy [13], and to attention that traditional medicine emphasizes the abortifacient potential of this plant and therefore discourages pregnant women from eating it, the effect of *A. fascicolifolius* manna investigated on the abortifacient creation. *A. fascicolifolius* manna as a byproduct is the result of the activity of an insect in the order Coleoptera on these shrubs. According to traditional medicine, this plant has several properties including an earache- and joint pain-relieving and skin patch and wart treating [14-16]. *A. fascicolifolius* manna contains glycosides, saponins and alkaloids [13, 17]. Because certain changes occur in the levels of estrogen, progesterone LH and FSH in pregnancy and abortion, and *A. fascicolifolius* may contribute to induced abortion through influencing the levels of these hormones, this study was conducted to aim determining abortifacient *A. fascicolifolius* manna hydroalcoholic extract effect on estrogen, progesterone, LH and FSH levels in BALB/c mice [18, 19].

## 2. Materials and methods

### 2.1. Preparation of manna extract

*A. fascicolifolius* manna samples were collected from the heights of Geno, Hormozgan province, with an altitude of 300 m, and botanically identified as *A. fascicolifolius* manna and assigned the herbarium no. 721 at the Medical Plants Research Center of Shahrekord University of Medical Sciences (SKUMS). The samples were dried and then pulverized. Extraction was conducted with 50% ethanol. The resulting extract was filtered with Whatman filter paper grade 1. Then, the concentrated extract was incubated at 37°C to dry.

### 2.2. Determining total saponin content of the extract

First, 100 mg of the plant powder was mixed with 10 ml of distilled water and then finely stirred for 30 seconds. The formation of persistent foam 30 minutes later was considered to represent the presence of saponin. To determine saponin content, 25 g of the plant powder was extracted using 250 ml of 50% ethanol under reflux for 3 hours. The mixture was filtered by a Whatman filter paper grade 2 and concentrated under vacuum. The resulting aqueous extract was extracted three times with equal amounts of n-butanol. Afterward, the n-butanol phase was decanted and concentrated by a rotary evaporator under vacuum. The residue was freezedried and its weight considered total saponin content [20].

### 2.3. Determination of total phenolic and flavonoid content of the extract

The total phenolic content of the extract was assayed by the Folin–Ciocalteu method. The total flavonoid content in *A. fasciculifolius* extract was determined using the aluminium chloride colourimetric method. Total phenolic and total flavonoid content was calculated from the calibration curve, and the results were expressed as mg of gallic acid equivalent per g dry weight and as mg rutin equivalent per g dry weight, respectively [21].

### 2.4. Determination of antioxidant capacity of the extract

The antioxidant activity of *A. fasciculifolius* extract was determined by 1, 1-diphenyl-2-picrylhydrazyl (DPPH) free radical scavenging assay. The percent inhibition of DPPH free radicals was calculated from (A control-A sample/Control) × 100, where Ablank is the absorbance of the control reaction, and A sample is the absorbance of the solution in the presence of plant extract. The concentration causing 50% inhibition of DPPH free radicals (IC_50_) was calculated from the regression equation for the concentration of extract and percentage inhibition [22].

### 2.5. Animal study

The protocol of this experimental study conforms to the Guide for the Care and Use of Laboratory Animal and the ethical guidelines of the SKUMS Bioethics Committee (approval no.: IR.SKUMS.-REC.1394.231). In this study, a total of 70 female BALB/c mice aged 8-12 weeks and weighing 25-30g were housed at (21 ± 2) °C and 12-h light/dark cycle as they had free access to the same food and water. The mice were assigned to seven groups of equal numbers as follows:

Group 1: Nonpregnant untreated; group 2: Nonpregnant *A. fascicolifolius* manna extract (400 mg/kg)-treated; Groups 3-5: Pregnant, *A. fascicolifolius* manna extract (400, 800 and 1200 mg/kg)-treated (The extract was intraperitoneally administered on 7-14 days); group 6: Pregnant, distilled water treated; and group 7: Pregnant, untreated.

**Fig. 1:**
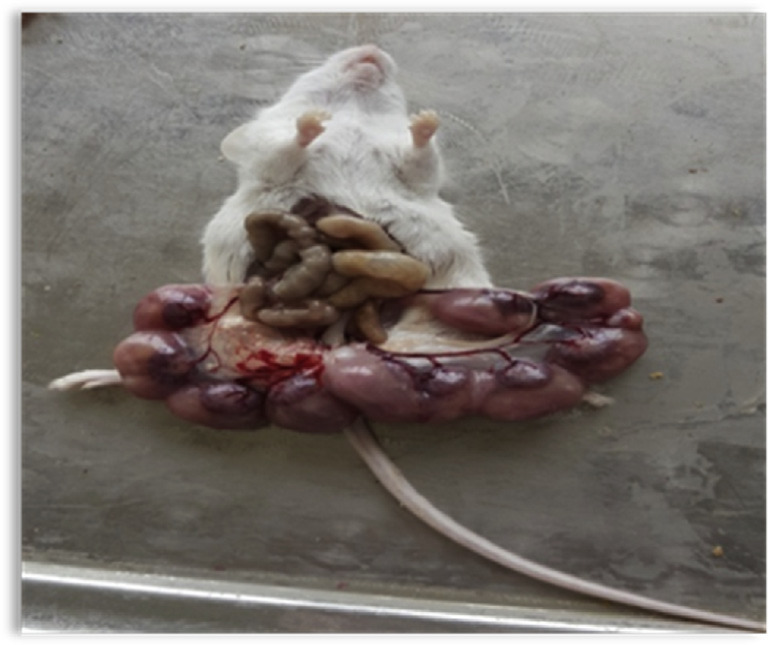
Beaded fallopian tubes (the photo was taken by author).

Because the pregnancy period in mouse is 21 days, on days 18 and 19 of pregnancy blood samples were collected from hearts according to the ethical guidelines under general anesthesia and serum levels of estrogen, progesterone, LH and FSH measured by the ELISA. Then, the cesarian section was performed on the mice; their beaded fallopian tubes (Fig. 1) removed and then resorbed fetuses in these tubes, representing the number of aborted fetuses, counted [23].

### 2.6. Measuring FSH, LH, Progesterone and Estrogen level in serum

After the blood samples were collected from the hearts, they were transferred to microtubes and serum samples isolated by centrifuging. Then, the serum levels of estrogen, progesterone, FSH and LH were measured by a standard ELISA kit (Shanghaicristal Day Biotech Co., China). First, standard solution was diluted and then 40 λ of each serum sample was added to the ELISA kit wells. Then, 10 λ of the specific antibodies of the serum parameters and 50 Landa of streptavidin solution was added to the wells. The ELISA kit was incubated at 37°C for 1 hour and then the wells emptied. The washing solution was diluted to 1.30 and 200 λ of the resulting washing solution added to each well in triplicate. Then, 50 λ of chromogen a solution and 50 λ of chromogen B solution were added to the wells and then the ELISA kit incubated at 37°C for 10 minutes. Finally, a stop solution was added to the wells. Then, the ELISA kit was placed into an ELISA reader to yield the data prints.

### 2.7. Statistical Analyses

The normal distribution of data was investigated by the Shapiro-Wilks test. Data were presented as mean ± SD (standard deviation) for normally distributed variables and as median (IQR: inter-quartile range) for non-normal distributed variables. ANOVA followed by the least significant difference (LSD) was used to compare the normally distributed variables between the groups and the Kruskal-Wallis test followed by Dunn's test to compare the non-normally distributed variables between the groups. Statistical significance was defined as P < 0.05 and analysis was performed by SPSS version 23.

## 3. Results

### 3.1. Phytochemical contents of the extract

The saponin content of the dried plant (25 g) was approximately 32 ± 2 %w/w. The total phenolic content was 36.35 ± 1.58 mg of gallic acid equivalent per g dry weight while the content of flavonoids was negligible. The antioxidant capacity of *A. fasciculifolius* extract by DPPH method was determined and the IC50 value of the extract was found to be 223.57 ± 10.07 mg/ml.

### 3.2. Effect of A. fascicolifolius manna extract on FSH and Progesterone

The levels of FSH increased significantly in the pregnant extract (400, 800 and 1200 mg/kg)-treated groups compared to the pregnant untreated group (p < 0.001), but this increase was not significant in the nonpregnant extract (400 mg/kg)-treated group compared to the nonpregnant untreated group (p > 0.05). The progesterone level decreased significantly in the pregnant extract (400, 800 and 1200 mg/kg)-treated groups compared to the pregnant untreated group (p < 0.001), but this hormone decrease was not significant in the nonpregnant extract (400 mg/kg)-treated group compared to the nonpregnant untreated group (p > 0.05) (Table 1).

### 3.3. Effect of A. fascicolifolius manna extract on LH, and Estrogen Level and abortion percentage

Abortion percentage was significantly different between the pregnant extract (400, 800 and 1200 mg/kg)-treated groups and the pregnant untreated group (p < 0.01), but not between the distilled water treated and pregnant untreated groups (p > 0.05). LH level increased significantly in the pregnant extract (400, 800 and 1200 mg/kg)-treated groups compared to the pregnant untreated group (p < 0.001), but this increase was not significant in the nonpregnant extract (400 mg/kg)-treated group compared to the nonpregnant untreated group (p < 0.05). Estrogen level was not significantly different between the pregnant extract (400, 800 and 1200 mg/kg)-treated groups compared to the pregnant untreated group as well as between the nonpregnant extract (400 mg/kg)-treated group and the nonpregnant untreated group (p > 0.05) (Table 2).

## 4. Discussion

Studies have indicated that women tend to consume herbal drugs because it is commonly believed that herbal drugs have many benefits and are not harmful to the body; therefore, most people are willing to use medicinal herbs, rather than chemical drugs, to cure diseases [24, 25]. Most pregnant women assume that nature-based drugs cause no drug interactions, fetal, and maternal side effects and therefore turn to self-medication with them [26]. Around 205 million pregnancies occur each year worldwide, over one-third of which is unplanned. Around one-fifth of these pregnancies lead to intentional abortion. Most intentional non-therapeutic abortions are due to unplanned pregnancies [1]. The intentional abortion has a long history and has been performed using various techniques such as the abortifacient herbs and the use of sharp implements and other traditional methods [27, 28].

Therapeutic intentional abortion occurs if there are maternal or fetal indications [29]. Abortion can occur spontaneously, which is called unintentional abortion [30]. One of the environmental causes of unintentional abortions is the use of medicinal plants such as *A. fascicolifolius* manna. To the best of our knowledge, no study has yet been conducted to investigate the abortifacient property of *A. fascicolifolius* manna. In the current study, we observed a significant difference in abortion percentage between the groups treated with *A. fascicolifolius* manna extract at 400, 800 and 1200 mg/kg and the pregnant untreated group and the abortion percentage increased with increasing the extract dose. The current study also showed that *A. fascicolifolius* manna extract probably could cause abortion.

Besides that, progesterone level decreased and FSH and LH levels increased significantly in the pregnant extract-treated groups compared to the pregnant untreated group; although *A. fascicolifolius* manna extract caused an increase in estrogen, this increase was not statistically significant. Because the progesterone level in the pregnant *A. fascicolifolius* manna extract-treated groups decreased significantly compared to the pregnant untreated group, and progesterone is required for maintaining pregnancy and implantation, and its reduction leads to a decrease in fetal growth and resorption, then probably decrease in progesterone level can be one of the mechanisms of significant increase in abortion in the extract-treated groups.

The compounds of *A. fascicolifolius* manna have not yet been fully and precisely identified, but phytochemical investigations on other mannas have confirmed the presence of compounds as well as saponin and alkaloids, and quantitative studies should also be conducted to precisely detect and isolate the compounds of *A. fascicolifolius* manna [13, 31]. Some alkaloids, such as berberine in barberry fruit, pass easily into organic cations of the cell membrane and form a complex with the DNA molecule and cause structural changes in it, which leads to a change in the regulatory activities of the genes as well as impairments in the differentiation pathways [32]. Recent Studies on antimicrobial and antineoplastic effects of alkaloids have demonstrated that these compounds inhibit oxygen supply by the cell, and oxygen uptake complete inhibit causes cell death and stops or restricts fetal growth [33, 34]. Studies have been demonstrated that saponin exerts abortifacient, anti-zygote and anti-implantation properties [35]. This compound can also exert teratogenic effects and therefore cause abortion or fetal death in rabbit and mouse [36].

Besides that, the study of Dollahite *et al*. showed that saponin could increase uterine contractions [37]. The phytochemical investigations in the present study demonstrated the significant and acceptable amount of total saponin in *A. fascicolifolius* manna extract, contrary to lack of phenol and flavonoid in the extract. Fetal resorption can be attributed to the anti-fetus effects of the alkaloid and saponin present in *A. fascicolifolius* manna. In this study, there was no significant difference in abortion percentage between the distilled water treated and control group and therefore the stress due to taking mice and the extract administration did not contribute to increased abortion.

Our results showed that the progesterone level decreased significantly and dose-dependently in the pregnant treated groups with *A. fascicolifolius* manna extract compared to the pregnant untreated group. It has been reported that alkaloids inhibit the activity of aromatase (the key enzyme in the synthesis of steroids) and thus reduce the synthesis of steroid hormones, including progesterone [38]. Also, saponin decreases the secretion of progesterone by reducing cholesterol, an essential ingredient for steroid hormone synthesis [39].

The alkaloid and saponin in *A. fascicolifolius* manna can be one of the mechanisms of a significant decrease in progesterone level in the pregnant mice treated with its extract. The estrogen level in the pregnant treated groups with *A. fascicolifolius* manna at different doses increased compared to the pregnant untreated group in a dose-dependent response yet insignificantly. Probably, the abortifacient effects of this extract may be due to different mechanisms from those leading to changes in estrogen levels. The levels of FSH and LH increased significantly in the pregnant treated groups with *A. fascicolifolius* manna at different doses compared to the pregnant untreated group, with the highest FSH and LH levels observed in the pregnant group receiving this extract at 1200 mg/kg. Probably, decreased the level of progesterone in the treated group with this manna extract is likely to stimulate the secretion of GnRH from the hypothalamus through negative feedback, which in turn stimulates the synthesis of pituitary gonadotropins [40, 41].

The changes in the levels of estrogen, progesterone, FSH and LH in the nonpregnant mice treated with *A. fascicolifolius* manna extract to compare with the nonpregnant untreated mice were similar to pregnant mice; more clearly, progesterone level decreased and estrogen, FSH and LH levels increased in the mice receiving *A. fascicolifolius* manna extract yet insignificantly. These insignificant hormonal changes in the nonpregnant *A. fascicolifolius* manna extract-treated mice can be attributed to the lower sensitivity of estrogen- and progesterone-secreting cells in the ovary compared to that of placental syncytial trophoblasts to *A. fascicolifolius* manna compounds. Therefore, it is necessary to conduct additional studies to investigate the effects of *A. fascicolifolius* manna extract at higher doses on the levels of progesterone, estrogen, FSH and LH in nonpregnant mice.

Regarding the significant percentage of abortion in the mice receiving *A. fascicolifolius* manna extract, we can argue that probably this *A. fascicolifolius* manna has an abortifacient effect and this abortion increase due to decreased progesterone level. The presence of certain compounds such as saponin and alkaloids in this extract may be the cause of the decrease in progesterone levels. This argument needs further investigation. Pregnant women are recommended to consume this plant more cautiously. *A. fascicolifolius* manna can also be used to induce abortion in women with indications for therapeutic abortion.

## Figures and Tables

**Table 1:** Effects of A. fascicolifolius manna on FSH and Progesterone in the different groups.

Variable Groups	FSH (pmol/l)	Progesterone (pmol/l)
	M ± SD	M ± SD
Nonpregnant untreated	12/267 ± 0/567	11/668 ± 0/311
Nonpregnant extract (400 mg/kg)-treated	13/304 ± 0/705	11/530 ± 0/277
Pregnant extract (400 mg/kg)-treated	11/117 ± 0/398	16/240 ± 0/503
Pregnant extract (800 mg/kg)-treated	11/400 ± 0/303	15/963 ± 0/452
Pregnant extract (1200 mg/kg)-treated	0/545 ± 11/485	15/761 ± 0/189
Pregnant distilled water treated	0/212 ± 12/200	10/660 ± 0/151
Pregnant untreated	0/216^a^± 10/266	16/906 ± 0/441^b^
Signification(P)	0/001	0/001

a Significant difference in FSH level compared to *Astragalus fascicolifolius* manna extract (400, 800 and 1200 mg/ml)-treated, distilled water treated and nonpregnant untreated groups (*p* < 0.001).

b Significant difference in progesterone level compared to *A*. *fascicolifolius* Manna extract (400, 800 and 1200 mg/ml)-treated, distilled water treated and nonpregnant untreated groups (*p* < 0.001).

**Table 2:** Effects of A. fascicolifolius manna on LH and estrogen levels and abortion percentage in the different groups.

Variable Groups	LH (pmol/l)	(pmol/l) estrogen	abortion percentage
	Med (IQR)	Med (IQR)	Med (IQR)
Nonpregnant untreated	2/950 (2/800-3/050)	28/960 (28/300-30/125)	
Nonpregnant extract (400 mg/kg)-treated	3/015 (2/890-3/275)	32/650 (30/122-30/125)	
Pregnant extract (400 mg/kg)-treated	4/610 (4/575-4/667)	35/875 (35/550-36/325)	0/190 (0/097-0/200)
Pregnant extract (800 mg/kg)-treated	4/500 (4/460-5/070)	39/150 (38/050-41/250)	0/236 (0/200-0/279)
Pregnant extract (1200 mg/kg)-treated	4/700 (4/640-5/560)	40/600 (39/000-41/700)	0/300 (0/250-0/400)
Pregnant distilled water treated	2/950 (2/145-3/300)	35/500 (34/650-35/650)	0/000 (0/000-0/091)
Pregnant untreated	2/300 (1/975-2/340)^a^	36/150 (35/250-36/375)^b^	0/000 (0/000-0/016)^c^
Signification (P)	0/001	0/001	0/001

a Significant difference in LH level compared to *Astragalus fascicolifolius* manna extract (400, 800 and 1200 mg/ml)-treated and nonpregnant untreated groups (*p* < 0.001).

b Significant difference in estrogen level compared to *A*. *fascicolifolius* manna extract (400, 800 and 1200 mg/ml)-treated groups (*p* < 0.01).

c Significant difference in abortion percentage compared to *A*. *fascicolifolius* manna extract (400, 800 and 1200 mg/ml)-treated groups (*p* < 0.01).
